# Supplementary material for: A Pilot Study on the Effects of Transcranial Direct Current Stimulation on Brain Rhythms and Entropy during Self-Paced Finger Movement using the Epoc Helmet
Source: Front Hum Neurosci. 2017 Apr 25;11:201. doi: 10.3389/fnhum.2017.00201 (PMC5408787; doi:10.3389/fnhum.2017.00201)
Supplement: Supplementary file 1 [file Data_Sheet_1.zip › Complementary_results/Power-Spectral-Entropy_complete_EEG/PSE_complete-EEG_AF4.pdf]

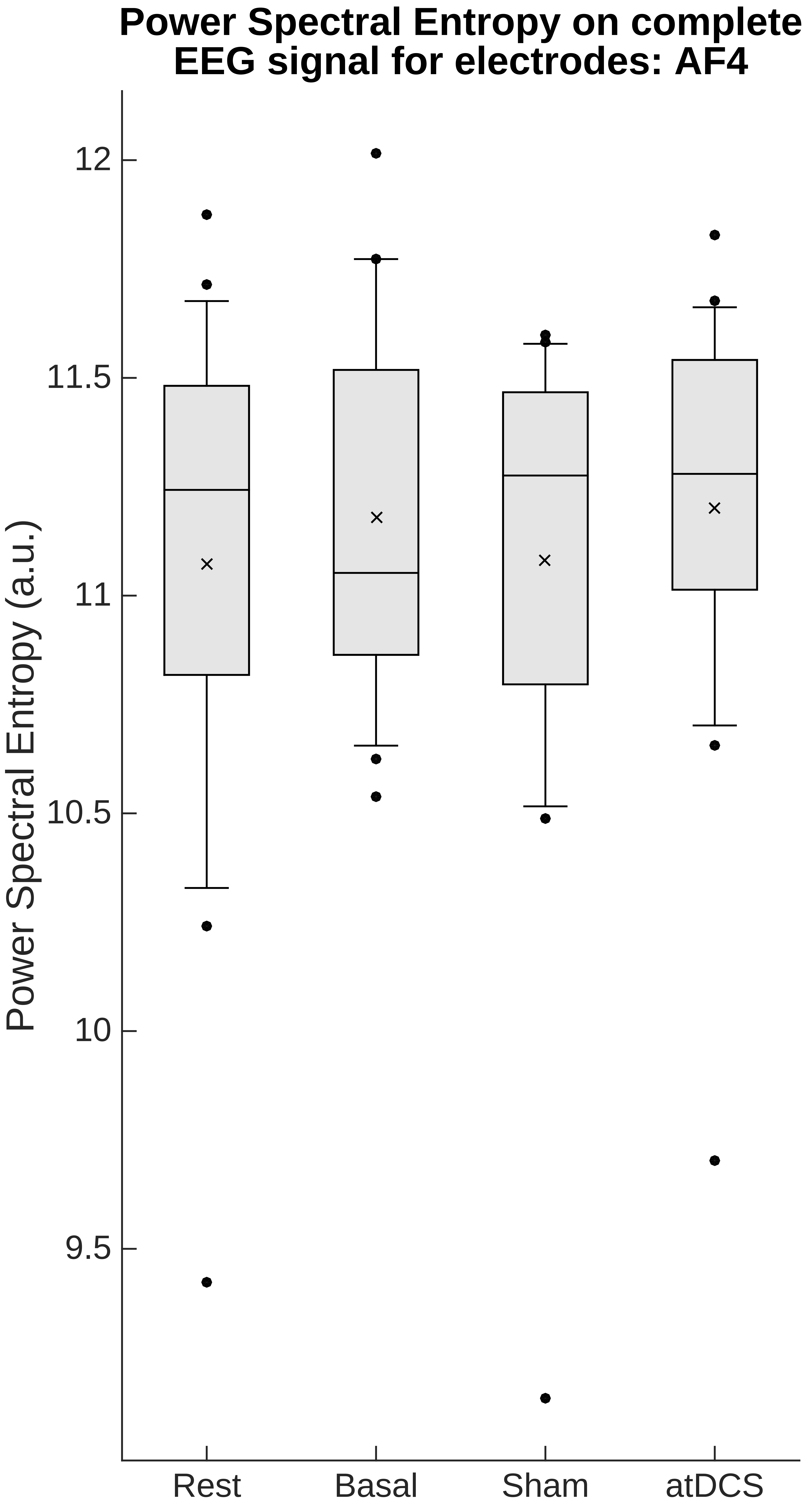
Power Spectral Entropy on complete
EEG signal for electrodes: AF4
Power Spectral Entropy (a.u.)
12
11.5
11
10.5
10
9.5
Rest
Basal
Sham
atDCS
